# Pacritinib inhibits glucose consumption in squamous cell lung cancer cells by targeting FLT3

**DOI:** 10.1038/s41598-023-28576-2

**Published:** 2023-01-25

**Authors:** Chiara Ghezzi, Bao Ying Chen, Robert Damoiseaux, Peter M. Clark

**Affiliations:** 1 Crump Institute for Molecular Imaging, University of California, Los Angeles, Box 951770, Los Angeles, CA 90095 USA; 2 Department of Molecular and Medical Pharmacology, University of California, Los Angeles, Los Angeles, CA 90095 USA; 3 California NanoSystems Institute, University of California, Los Angeles, Los Angeles, CA 90095 USA; 4 Eli and Edythe Broad Center of Regenerative Medicine and Stem Cell Research, University of California, Los Angeles, Los Angeles, CA 90095 USA

**Keywords:** Cancer imaging, Cancer metabolism, Cancer therapy, Lung cancer

## Abstract

Squamous cell lung cancer maintains its growth through elevated glucose consumption, but selective glucose consumption inhibitors are lacking. Here, we discovered using a high-throughput screen new compounds that block glucose consumption in three squamous cell lung cancer cell lines and identified 79 compounds that block glucose consumption in one or more of these cell lines. Based on its ability to block glucose consumption in all three cell lines, pacritinib, an inhibitor of FMS Related Receptor Tyrosine Kinase 3 (FLT3) and Janus Kinase 2 (JAK2), was further studied. Pacritinib decreased glucose consumption in squamous cell lung cancer cells in cell culture and in vivo without affecting glucose consumption in healthy tissues. Pacritinib blocked hexokinase activity, and Hexokinase 1 and 2 mRNA and protein expression. Overexpression of Hexokinase 1 blocked the ability of pacritinib to inhibit glucose consumption in squamous cell lung cancer cells. Overexpression of FLT3 but not JAK2 significantly increased glucose consumption and blocked the ability of pacritinib to inhibit glucose consumption in squamous cell lung cancer cells. Additional FLT3 inhibitors blocked glucose consumption in squamous cell lung cancer cells. Our study identifies FLT3 inhibitors as a new class of inhibitors that can block glucose consumption in squamous cell lung cancer.

## Introduction

Elevated glucose consumption in cancer including squamous cell lung cancer is driven by oncogenes and occurs in the vast majority of cancers as demonstrated by [^18^F]fluorodeoxyglucose [(^18^F)FDG] positron emission tomography (PET) imaging^1–3^. Squamous cell lung cancer is a prevalent subtype of non-small cell lung cancer with a low five-year survival rate and limited targeted therapy options for those with advanced disease^4–7^. Squamous cell lung cancer cells have elevated protein levels of the Solute Carrier Family 2 Member 1 (SLC2A1; also known as Glucose Transporter 1 or GLUT1) glucose transporter that results in elevated glucose consumption compared to normal lung tissue and in a strong dependency on elevated glucose consumption for their growth and survival^3,8,9^. Targeting glucose consumption in squamous cell lung cancer–alone or in combination with other therapies–can limit cell growth and induce apoptosis^3,9,10^. Drugs that selectively block glucose consumption in squamous cell lung cancer could function as single agents or in combination therapies to treat this deadly disease. However, there are currently no therapeutic approaches to selectively target glucose consumption in squamous cell lung cancer.

Metabolism remains an important target in cancer^11–13^, but identifying therapeutic approaches to selectively target cancer glucose consumption has proven challenging. Most studies that target glucose consumption in cancer have used the molecule 2-deoxyglucose (2-DG), which at high concentrations can target the hexokinase enzymes that metabolize glucose^3,10,14^. However, these enzymes are found in most normal cells, and 2-DG has a small therapeutic window for cancer over normal cells, causing significant side effects^15,16^. Similar approaches to directly target the SLC2A (GLUT) family of glucose transporters or enzymes in the glycolysis pathway are being tested but similar challenges are expected^17^.

Recent studies suggest that targeting pathways that regulate glucose consumption in cancer cells but *not* in normal cells can be an effective strategy for selectively targeting glucose consumption in cancer while sparing healthy tissue^18–20^. Such pathways exist because elevated glucose consumption in cancer is often driven by the same genetic alterations that induce and are unique to cancer^1^. We recently developed a high-throughput assay for measuring glucose consumption^18^. Here we use this assay to screen 3555 small molecules against three squamous cell lung cancer cell lines to identify new inhibitors of glucose consumption and to further study one inhibitor, pacritinib (Supplementary Figure. 1).

## Results

### A high-throughput screen discovers novel inhibitors of squamous cell lung cancer glucose consumption

To discover new small molecule inhibitors of squamous cell lung cancer glucose consumption, we used a high-throughput glucose consumption assay that we recently developed^18^. In this assay, cells are treated with compounds, and glucose consumption is measured by quantifying 2-DG-6-phosphate levels in cells treated with 2-DG. Because we remove the compounds from the cells prior to assaying glucose consumption, our approach favors the identification of compounds that alter pathways that regulate glucose consumption rather than direct inhibitors of the enzymes involved in glucose consumption. We studied squamous cell lung cancer glucose consumption using two squamous cell lung cancer cell lines–SK-MES-1 and H520 cells–and a cell line with mixed squamous and adenocarcinoma characteristics–H596 cells. Clinically, squamous cell lung cancer is characterized by alterations in p53, kelch-like ECH-associated protein 1 (KEAP1), phosphatase and tensin homolog (PTEN)/phosphatidylinositol-3-kinase (PI3K), cyclin dependent kinase inhibitor 2A (CDKN2A), and RB1^21^. All three of the cell lines we studied have inactivating mutations in p53, H596 cells have mutations in phosphatidylinositol 4,5-bisphosphate 3-kinase catalytic subunit alpha isoform (PIK3CA) and RB1, and H520 cells have a mutation in CDKN2A^22^.

**Figure 1:**
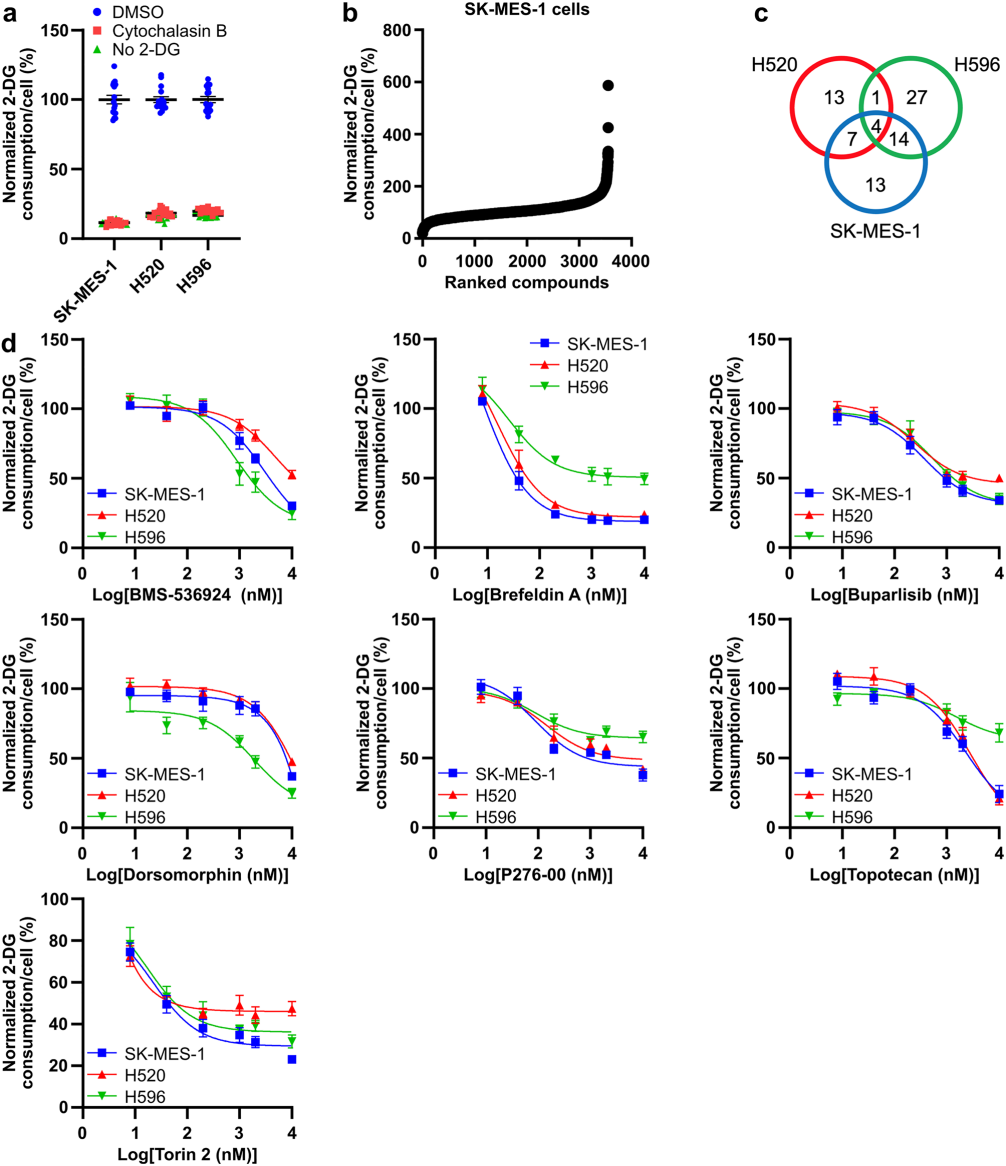
A high-throughput screen identifies small molecule inhibitors of squamous cell lung cancer glucose consumption. (**a**) Glucose consumption in SK-MES-1, H520, and H596 cells, as determined using a high-throughput glucose consumption assay that measures 2-DG-6-phosphate levels in 2-DG treated cells. Cells were treated with DMSO, Cytochalasin B, or without 2-DG. (**b)** A waterfall plot of glucose consumption in SK-MES-1 cells treated with 3555 compounds. (**c**) The number of unique and shared compounds that inhibit glucose consumption by > 50% in SK-MES-1, H520, and H596 cells. (**d**) Dose–response curves of glucose consumption in SK-MES-1, H520, and H596 cells treated with BMS-536924, Brefeldin A, buparlisib, dorsomorphin, P276-00, topotecan, and Torin 2.

For a high-throughput assay to yield interpretable results, it needs to have a *Z*-factor of > 0.5 for the cell lines tested. The *Z*-factor incorporates data on the dynamic range and variability of the assay. We determined the *Z*-factor of our assay in the SK-MES-1, H520, and H596 cells. The cells were treated with 2-DG and vehicle, 2-DG and the GLUT transporter inhibitor Cytochalasin B, and without 2-DG; and glucose consumption was measured using our high-throughput assay (Fig. 1a). Cells treated with 2-DG and Cytochalasin B or without 2-DG served as separate negative controls. When the cells were treated with 2-DG and Cytochalasin B, the assay yielded a Z-factor of 0.54, 0.60, and 0.60 for SK-MES-1, H520, and H596 cells, respectively. When the cells were treated without 2-DG, the assay yielded a Z-factor of 0.54, 0.63, and 0.61 for SK-MES-1, H520, and H596 cells, respectively. These results demonstrated that all three cell lines could be used to identify inhibitors of glucose consumption using our high-throughput glucose consumption assay in a high-throughput screen.

SK-MES-1, H520, and H596 cells were screened against 3555 bioactive small molecules from four small molecule libraries including the Prestwick library of mainly FDA-approved compounds, the Selleck Chemicals kinase inhibitors library, the LOPAC library of pharmacologically active compounds, and the NIH clinical collection. Glucose consumption per cell was measured 24 h later (representative waterfall plot of the results for SK-MES-1 cells is shown in Fig. 1b). From our high-throughput screen in which each compound was only tested once, we identified an average of 53 small molecules per cell line that inhibited glucose consumption by > 50% in our assay. Replication experiments with these small molecules yielded a 70% reconfirmation rate and 38, 25, and 46 confirmed small molecule inhibitors of glucose consumption in SK-MES-1, H520, and H596 cells, respectively (Supplementary Table 1).

Of the confirmed inhibitors of glucose consumption identified, 22 inhibited glucose consumption by > 50% in two of the cell lines, and 4 inhibited glucose consumption by > 50% in all three of the cell lines (Fig. 1c). In total, this yielded 79 unique compounds for further study. Less than half of the compounds we identified as inhibitors of squamous cell lung cancer glucose consumption also inhibited glucose consumption in any of the lung adenocarcinoma cell lines we previously studied^18^, indicating that most of the identified inhibitors of glucose consumption are unique to squamous cell lung cancer. The most efficacious small molecule inhibitors of squamous cell lung cancer glucose consumption include Brefeldin A, an inhibitor of the ADP-ribosylation factors involved in vesicle formation, the topoisomer inhibitors camptothecin and mitoxantrone, and kinase inhibitors SC-1, PD-1666285, and ponatinib.

We further validated our results and determined the potency for inhibiting glucose consumption of a subset of the compounds by studying the dose–response relationship between these inhibitors and glucose consumption. We studied the insulin-like growth factor 1 receptor (IGF1R) inhibitor BMS-536924, Brefeldin A, the PI3K inhibitor buparlisib, the AMP-activated protein kinase (AMPK) and bone morphogenetic proteins (BMP) inhibitor dorsomorphin, the cyclin-dependent kinase (CDK) inhibitor P276-00, the topoisomerase inhibitor topotecan, and the mechanistic target of rapamycin (mTOR) inhibitor Torin 2 across a concentration range of 8 nM–10 μM (Fig. 1d). These inhibitors blocked glucose consumption across a range of potencies with IC_50_ values extending from low nM to mid μM. Brefeldin A and Torin 2 proved to be the most potent inhibitors of glucose consumption among the tested compounds, with an IC_50_ of 8 nM in SK-MES-1 cells and 7 nM in H520 cells, respectively. Collectively, our results yield a list of small molecule inhibitors of squamous cell lung cancer glucose consumption for further study.

### Pacritinib blocks glucose consumption in squamous cell lung cancer cells

**Figure 2:**
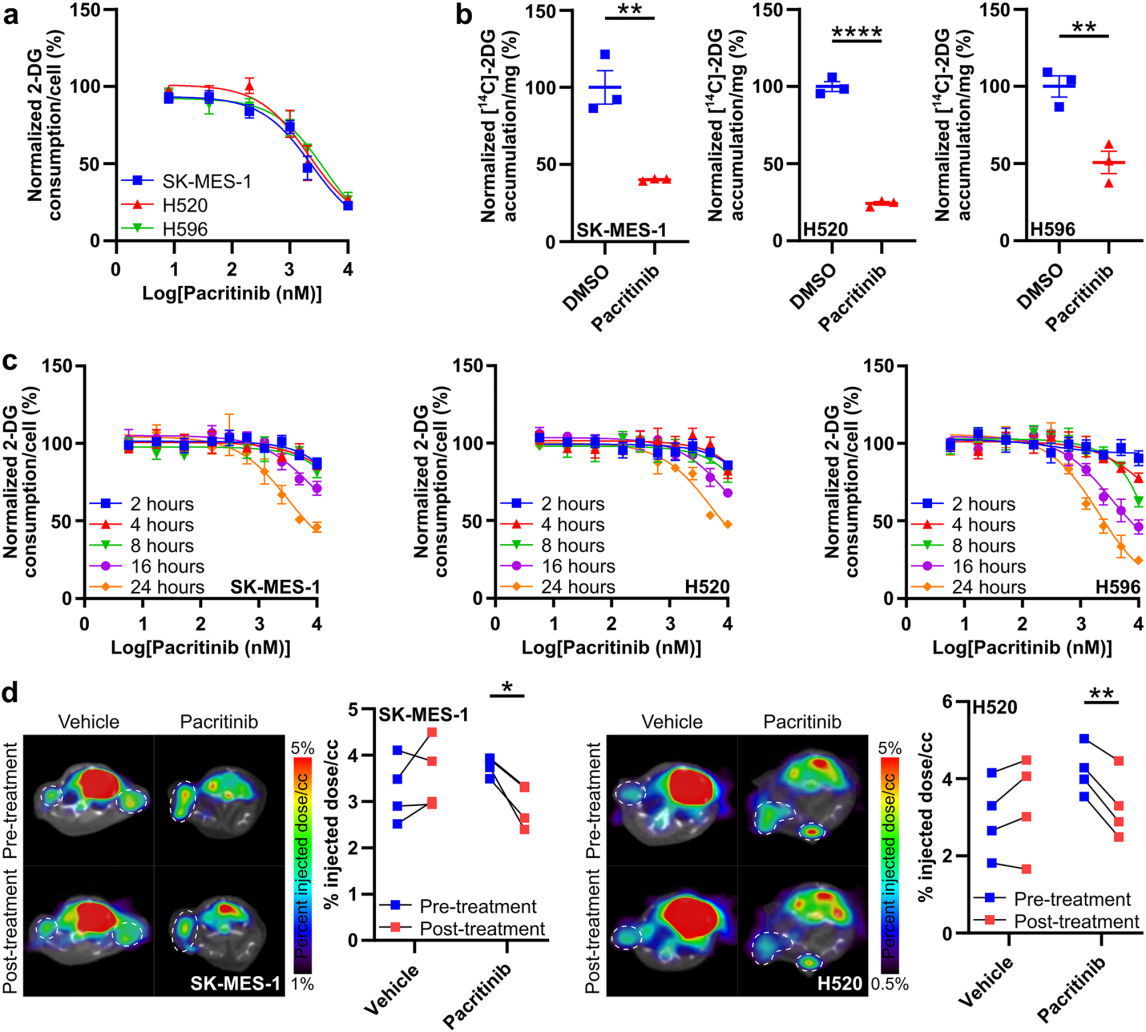
Pacritinib blocks glucose consumption in squamous cell lung cancer cell lines. (**a**) Glucose consumption as measured using a high-throughput assay in SK-MES-1, H520, and H596 cells treated with vehicle or pacritinib across a range of concentrations. (**b**) Glucose consumption as measured by [^14^C]2-DG accumulation in SK-MES-1, H520, and H596 cells treated with vehicle or pacritinib. (**c**) Glucose consumption in SK-MES-1, H520, and H596 cells treated with vehicle or pacritinib for 2, 4, 8, 16, and 24 h. (**d**) [^18^F]FDG PET images and quantification of [^18^F]FDG accumulation in SK-MES-1 and H520 xenografts pre- and post-treatment with vehicle or pacritinib. Xenograft tumors encircled in white dotted lines. *P < 0.05; **P < 0.01; ****P < 0.0001.

From our list of small molecule inhibitors of squamous cell lung cancer glucose consumption, we prioritized compounds (i) that decreased glucose consumption by > 50% in all three cell lines and by > 70% in at least one cell line we studied; (ii) that have been used in mice and people; (iii) that have not been previously linked to glucose consumption; (iv) that do not dramatically decrease cell growth at 24 h post-treatment; and (v) that inhibit glucose consumption with an IC_50_ value < 3 µM in at least one cell line. The first four criteria led us to further investigate pacritinib, a janus kinase 2 (JAK2) and fms related receptor tyrosine kinase 3 (FLT3) inhibitor that has shown efficacy for the treatment of myelofibrosis^23,24^. Pacritinib inhibited glucose consumption with an IC_50_ of 2.3, 2.1, and 4.0 µM in SK-MES-1, H520, and H596 cells, respectively (Fig. 2a), fulfilling our fifth criteria. This led us to study pacritinib in greater detail.

We further validated and expanded our results showing that pacritinib blocks glucose consumption. Pacritinib significantly decreased glucose consumption by 59.8 ± 0.5%, 75.7 ± 1.2%, and 49.3 ± 7.3% in SK-MES-1, H520, and H596 cells, respectively, when glucose consumption was assayed by measuring accumulated radioactivity in cells treated with [^14^C]2-DG (Fig. 2b). This provides additional evidence using an assay that measures glucose consumption with a readout that is different from the readout of our high-throughput assay that pacritinib blocks glucose consumption. In all three cell lines, 24 h of treatment was required to reach the full inhibitory effect of pacritinib on glucose consumption although significant effects on glucose consumption could be observed as early as 16 h after pacritinib treatment (Fig. 2c). To determine whether pacritinib blocks glucose consumption in squamous cell cancer cells in vivo, mice engrafted with SK-MES-1 and H520 xenografts were imaged with the glucose analogue PET radiotracer [^18^F]FDG before and after treatment with vehicle or pacritinib. Pacritinib significantly reduced glucose consumption by 22.8 ± 4.9% and 22.9 ± 4.1% in both the SK-MES-1 and H520 xenografts, respectively, *without* affecting glucose consumption in healthy tissues such as the brain, heart, and muscles (Fig. 2d). Collectively, this data demonstrates that pacritinib is a novel, selective inhibitor of glucose consumption for squamous cell lung cancer.

### Pacritinib blocks glucose consumption by decreasing Hexokinase 1 expression

**Figure 3:**
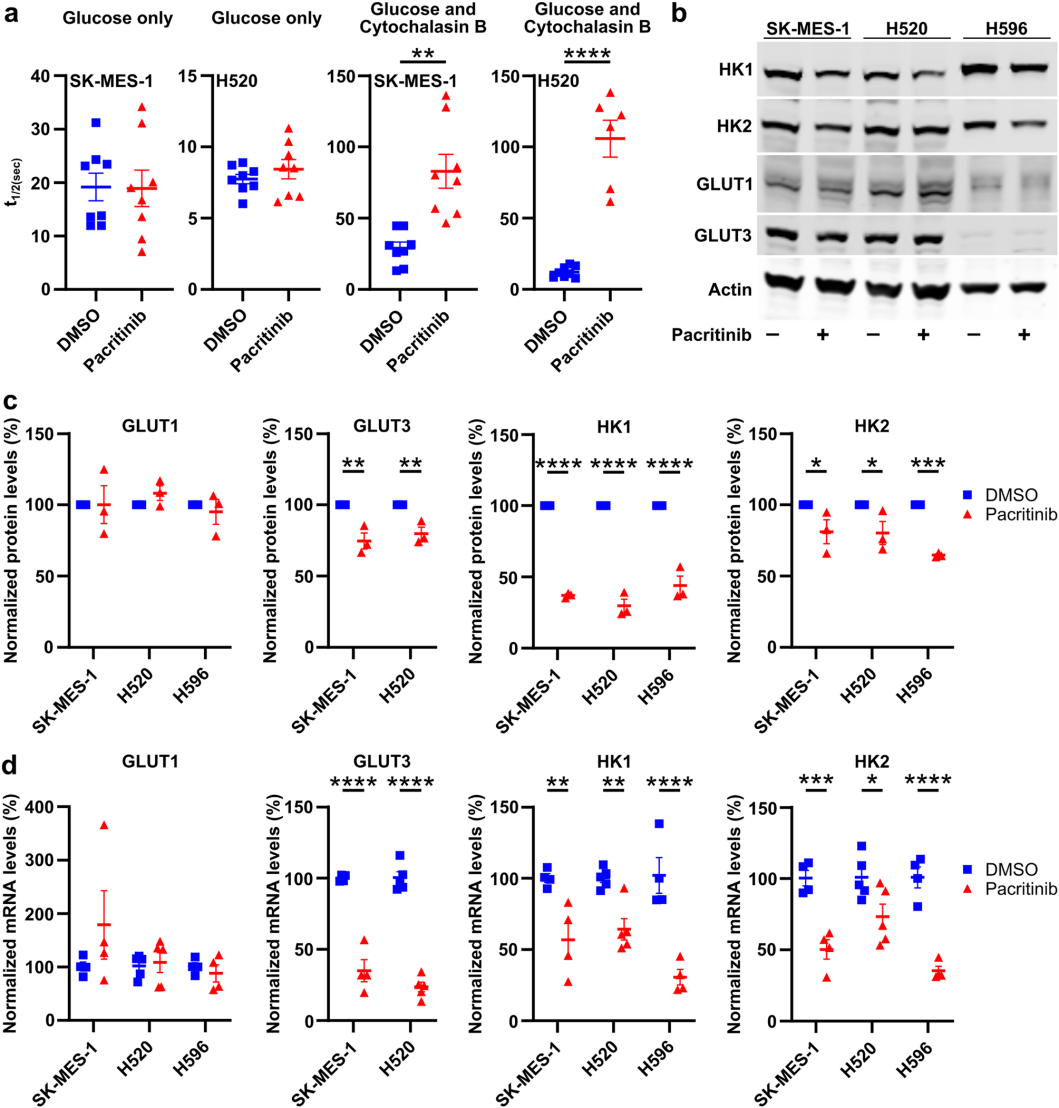
Pacritinib blocks hexokinase expression and activity. (**a**) The rate of change in yellow fluorescence for a FRET-based glucose reporter in SK-MES-1 and H520 cells treated with vehicle or pacritinib and incubated with glucose only or glucose and Cytochalasin B. (**b**) Representative immunoblots and (**c**) quantification of GLUT1, GLUT3, Hexokinase 1 (HK1), and Hexokinase 2 (HK2) protein levels in SK-MES-1, H520, and H596 cells treated with vehicle or pacritinib. Full-length, uncropped blots are presented in Supplementary Figure. 3. (**d**) mRNA levels of GLUT1, GLUT3, HK1, and HK2 in SK-MES-1, H520, and H596 cells treated with vehicle or pacritinib. *P < 0.05; **P < 0.01; ***P < 0.001; ****P < 0.0001.

The high-throughput assay we used to identify as well as the [^14^C]2-DG and [^18^F]FDG assays we used to confirm that pacritinib blocks glucose consumption all measure the combined activity of the GLUT glucose transporters and the hexokinase enzymes that phosphorylate glucose. Thus, for pacritinib to affect the readout of these assays, it must alter the levels or activity of one or more of these transporters or enzymes. To separately measure the activity of these two steps–glucose transport and glucose phosphorylation by the hexokinases–we used a fluorescence resonance energy transfer (FRET) construct that fluoresces yellow in the presence of glucose^25^. In cells expressing this FRET construct, the initial increase in yellow fluorescence when the cells are treated with glucose alone measures the rate of glucose transport while the decrease in yellow fluorescence when the cells are treated with glucose followed by Cytochalasin B measures the rate of glucose phosphorylation^25^. Pacritinib had no effect on the rate of increase in yellow fluorescence in SK-MES-1 and H520 cells treated with glucose alone, demonstrating that pacritinib does not affect glucose transport (Fig. 3a). However, pacritinib significantly decreased by 2.9 ± 0.4-fold and 8.7 ± 1.1-fold the rate of diminution of yellow fluorescence in both the SK-MES-1 and H520 cells, respectively, demonstrating that pacritinib decreases hexokinase levels or activity in these cells. H596 cells failed to express the FRET construct and could not be analyzed using this assay.

The best studied glucose transporters and hexokinase enzymes in cancer, including in squamous cell lung cancer, are GLUT1, SLC2A3 (also known as Glucose Transporter 3 or GLUT3), Hexokinase 1, and Hexokinase 2^26,27^. Pacritinib significantly decreased Hexokinase 1 mRNA levels by 43.2 ± 12.5%, 35.8 ± 7.5%, and 69.4 ± 5.4% and protein levels by 63.0 ± 0.9%, 70.3 ± 4.7%, and 56.0 ± 6.6% in SK-MES-1, H520, and H596 cells, respectively. Pacritinib also significantly decreased Hexokinase 2 mRNA levels by 49.7 ± 6.9%, 26.7 ± 8.9%, and 64.7 ± 3.2% and protein levels by 18.9 ± 8.4%, 19.7 ± 8.1%, and 35.2 ± 0.9% in SK-MES-1, H520, and H596 cells, respectively. H596 cells did not express GLUT3 at the mRNA or protein level, but pacritinib decreased GLUT3 mRNA levels by 64.9 ± 7.8% and 76.4 ± 3.4% and protein levels by 25.3 ± 5.6% and 20.2 ± 4.5% in SK-MES-1 and H520 cells, respectively (Fig. 3b,c,d). These results suggest that pacritinib blocks glucose consumption by inhibiting the expression of Hexokinase 1 and/or Hexokinase 2.

**Figure 4:**
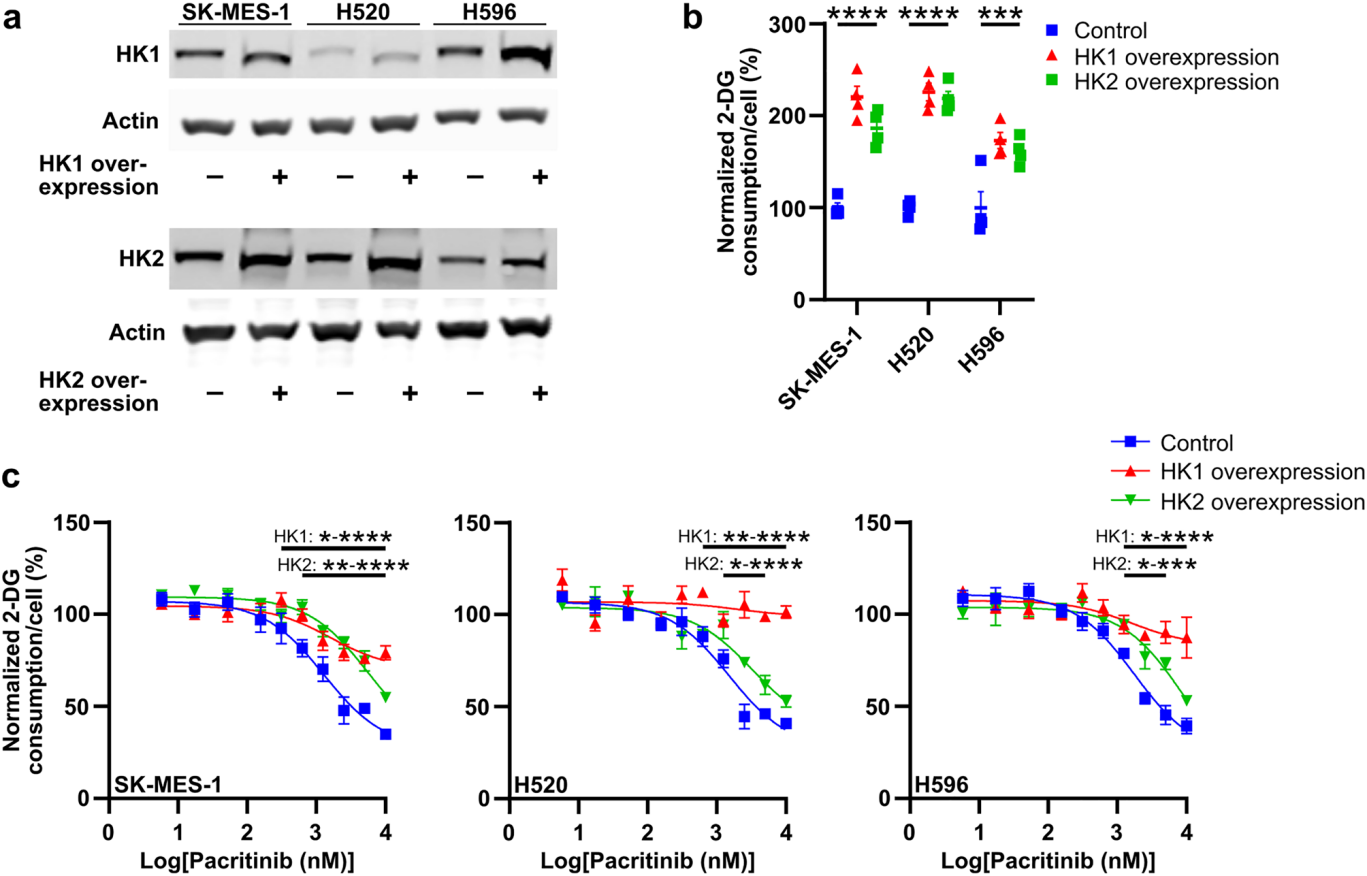
Hexokinase 1 overexpression rescues the inhibitory effect of pacritinib on glucose consumption. (**a**) Representative immunoblots of Hexokinase 1 (HK1) and Hexokinase 2 (HK2) protein levels in SK-MES-1, H520, and H596 cells transfected with HK1 and HK2 overexpression plasmids, respectively, or a control plasmid. Full-length, uncropped blots are presented in Supplementary Figure. 3. (**b**) Glucose consumption in control, HK1 overexpression, and HK2 overexpression SK-MES-1, H520, and H596 cells. (**c**) Glucose consumption in control, HK1 overexpression, and HK2 overexpression SK-MES-1, H520, and H596 cells treated with vehicle or pacritinib. *P < 0.05; **P < 0.01; ***P < 0.001; ****P < 0.0001.

To determine whether pacritinib blocks glucose consumption by inhibiting Hexokinase 1 or Hexokinase 2, we separately overexpressed Hexokinase 1 or Hexokinase 2 in SK-MES-1, H520, and H596 cells (Fig. 4a). Overexpression of Hexokinase 1 or Hexokinase 2 increased glucose consumption to a similar degree in all three cell lines (Glucose consumption with overexpression of Hexokinase 1 and Hexokinase 2, respectively, in SK-MES-1 cells: 220.2 ± 11.7% and 186.3 ± 9.6%, in H520 cells: 225.6 ± 9.5% and 218.5 ± 7.8%, in H596 cells: 172.8 ± 8.8% and 161.1 ± 7.3%; Fig. 4b). Both Hexokinase 1 and Hexokinase 2 overexpression significantly limited the ability of pacritinib to block glucose consumption. However, across all three cell lines, Hexokinase 1 overexpression more strongly blocked the ability of pacritinib to inhibit glucose consumption than Hexokinase 2 overexpression. In SK-MES-1, H520, and H596 cells, Hexokinase 1 overexpression rescued the inhibitory effect of pacritinib on glucose consumption by a maximum of 67.9 ± 4.9%, 102.3 ± 6.7%, and 79.1 ± 13.1%, respectively (Fig. 4c). This suggests a model in which pacritinib blocks glucose consumption in squamous cell lung cancer cell lines mostly by limiting Hexokinase 1 expression.

### Pacritinib blocks glucose consumption by targeting FLT3

**Figure 5:**
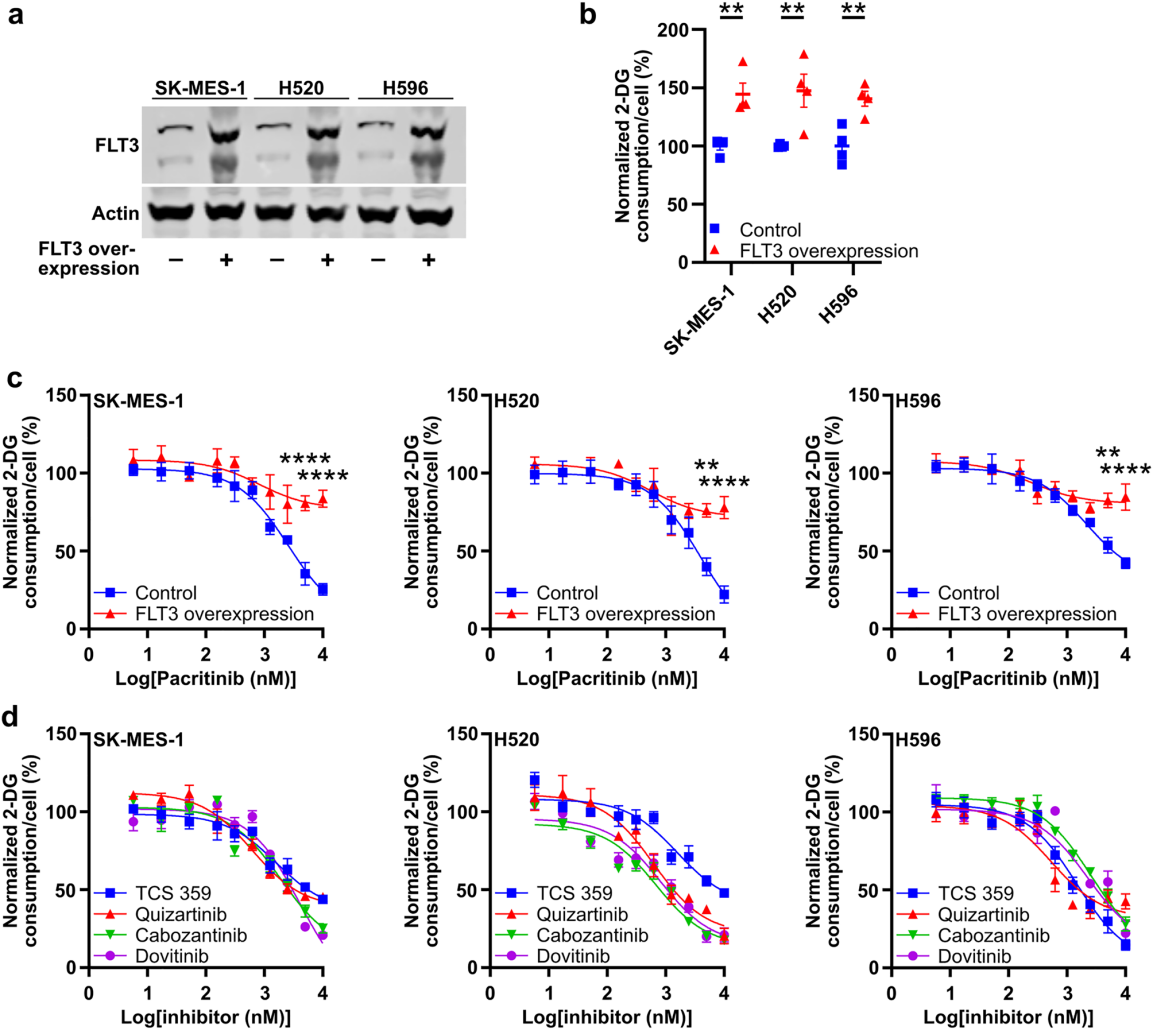
Pacritinib blocks glucose consumption through targeting FLT3. (**a**) Representative immunoblots of FLT3 in SK-MES-1, H520, and H596 cells transfected with control or FLT3 overexpression plasmids. Full-length, uncropped blots are presented in Supplementary Figure. 3. (**b**) Glucose consumption in control and FLT3 overexpression SK-MES-1, H520, and H596 cells. (**c**) Glucose consumption in control and FLT3 overexpression SK-MES-1, H520, and H596 cells treated with vehicle or pacritinib. (**d**) Glucose consumption in SK-MES-1, H520, and H596 cells treated with vehicle or additional FLT3 inhibitors. **P < 0.01; ****P < 0.0001.

Pacritinib is a potent inhibitor of JAK2 (in vitro IC_50_: 23 nM) and FLT3 (in vitro IC_50_: 22 nM)^24^. Overexpression of JAK2 in SK-MES-1, H520, and H596 cells had no effect on glucose consumption in untreated cells and no effect on the ability of pacritinib to inhibit glucose consumption (Supplementary Figure. 2). Additional JAK2 inhibitors XL019 and AZ960 also had no effect on SK-MES-1, H520, and H596 glucose consumption (Supplementary Figure. 2). However, overexpression of FLT3 in SK-MES-1, H520, and H596 cells increased glucose consumption in untreated cells by 44.5 ± 9.4%, 47.4 ± 14.3%, and 40.5 ± 6.4%. Overexpression of FLT3 also blocked the ability of pacritinib to inhibit glucose consumption in all three cell lines (Fig. 5a,b,c). FLT3 overexpression rescued the inhibitory effect of pacritinib on glucose consumption by a maximum of 78.0 ± 12.4%, 71.5 ± 18.8%, and 73.5 ± 9.3% in SK-MES-1, H520, and H596 cells, respectively. Additional small molecules that inhibit FLT3 with an in vitro IC_50_ < 50 nM including TCS 359, quizartinib, cabozantinib, and dovitinib all block glucose consumption in SK-MES-1, H520, and H596 cells (Fig. 5d). These data support a model in which pacritinib blocks glucose consumption in squamous cell lung cancer cells by inhibiting FLT3.

## Discussion

We show that pacritinib blocks glucose consumption in squamous cell lung cancer cells by inhibiting FLT3. Pacritinib was designed as a JAK2 and FLT3 inhibitor with the goal of developing a drug to target hematological malignancies driven by these kinases including myelofibrosis, lymphoma, and acute myeloid leukemia (AML)^28^. Structure–activity studies based on a macrocycle backbone yielded pacritinib, which has an in vitro IC_50_ of 23 nM and 22 nM for JAK2 and FLT3, respectively. Computational docking of a model compound based on the pacritinib backbone suggests that Asp698 on FLT3 makes a hydrogen bond to an ether oxygen in pacritinib, contributing to its strong and selective binding^29^. Consistent with the data showing pacritinib blocks FLT3 activity in vitro, in preclinical studies, pacritinib treatment of AML cells blocks FLT3 autophosphorylation as well as signaling pathways downstream of FLT3 including mitogen-activated protein kinase (MAPK) and PI3K signaling^30,31^. Pacritinib most potently inhibits JAK2 and FLT3 but like many other kinase inhibitors, at higher concentrations also inhibits additional kinases including colony stimulating factor 1 receptor (CSF1R) and c-KIT^32^.

Most studies on the role of FLT3 in cancer focus on activating FLT3 mutations including an internal tandem duplication in AML^33,34^. FLT3 in normal physiology has been mostly studies within the context of hematopoiesis^34^. A role for FLT3 in squamous cell lung cancer is not well-studied. No FLT3 mutations, translocations, or amplifications have been reported in the SK-MES-1, H520, and H596 cell lines that we studied here. Mutations in FLT3 are rare in tumor samples from patients with squamous cell lung cancer and most reported mutations are of unknown significance^35^. How FLT3 is activated to drive glucose consumption in our cell lines remains to be determined. However, the fact that we show that pacritinib blocks glucose consumption in squamous cell lung cancer in vivo without affecting glucose consumption in healthy tissues suggests that a role for FLT3 in driving glucose consumption is cancer specific. In the absence of activating mutations, FLT3 requires FLT3 ligand to be activated^33,34^. One possibility is that the mutations that are present in our cell lines such as inactivating p53 mutations lead to increased FLT3 ligand expression. That and other possibilities will be tested in future studies.

We show that pacritinib blocks glucose consumption by inhibiting Hexokinase 1 mRNA expression and protein levels, with the lower protein levels likely a consequence of the lower mRNA levels. We do not yet understand the mechanism by which FLT3 activates Hexokinase 1 mRNA expression. Like many receptor tyrosine kinases, FLT3 activates the RAS/MAPK kinase (MEK)/extracellular-signal-regulated kinase (ERK) and the PI3K/Protein Kinase B (AKT) pathways^33^. The PI3K/AKT pathway has been strongly linked to glucose consumption when activated by other receptor tyrosine kinases^36^, although specific mechanisms through which this pathway activates glucose consumption are likely receptor tyrosine kinase- and cancer-type specific. FLT3, when activated by an internal tandem duplication (FLT3-ITD), activates glucose consumption in acute myeloid leukemia cells^37^. In that case, FLT3-ITD expression activates AKT to phosphorylate Hexokinase 2, leading to increased Hexokinase 2 localization at the outer mitochondrial membrane where Hexokinase 2 is known to drive glucose consumption. Insofar as we find that FLT3 drives Hexokinase 1 mRNA and protein levels, a different mechanism is likely involved here and possibly one involving increased transcription of Hexokinase 1. Future studies will determine the specific pathway through which pacritinib, by blocking FLT3 activity, reduces Hexokinase 1 expression.

We show that Hexokinase 1 overexpression can rescue the inhibitory effect of pacritinib on glucose consumption, suggesting Hexokinase 1 as a major driver of elevated glucose consumption in squamous cell lung cancer cells and a potential target for additional therapeutic strategies. Although Hexokinase 1 can contribute to cancer, Hexokinase 2 is more frequently overexpressed in various cancers including colorectal, liver, cervical, and metastatic breast cancer and glioblastoma, and its overexpression is associated with a worse survival^38–42^. We do not know why Hexokinase 1 rather than Hexokinase 2 drives glucose consumption in the cell lines we studied. KRAS4A can interact with Hexokinase 1 and relieve glucose-6-phosphate-dependent allosteric inhibition of Hexokinase 1^43^ possibly enabling Hexokinase 1 to be a stronger driver of glucose consumption than Hexokinase 2 in certain cancers. The cell lines we studied all express wild-type KRAS, suggesting against this possibility, but we speculate that a similar mechanism driven by a different oncogene may function in squamous cell lung cancer cells. Hexokinase 1 is expressed in tissues throughout the body^44^, and directly targeting Hexokinase 1 would likely cause significant toxicities. Additional strategies to target Hexokinase 1 will have to follow the approach outlined here of targeting a cancer-specific pathway that drives Hexokinase 1 protein levels or activity rather than targeting Hexokinase 1 directly.

Using our high-throughput glucose consumption assay, we identified over 79 different small molecules out of the 3555 we screened that block glucose consumption in one or more of the three squamous cell lung cancer cell lines we studied. This is consistent with our studies in non-small cell lung adenocarcinoma cells where we identified 97 different small molecules that inhibited glucose consumption^18^ and suggests that a large number of different pathways converge to drive elevated glucose consumption in squamous cell lung cancer. However, the fact that only 5% of these compounds inhibited glucose consumption in all three squamous cell lung cancer lines and that there is < 50% overlap in the compounds identified from the squamous and adenocarcinoma cell lines suggest that these pathways are strongly dependent on the underlying mutational drivers of the cancer. It will be important going forward to identify those pathways driven by common cancer mutations to prioritize for study.

## Conclusions

In conclusion, we identify pacritinib as a strong inhibitor of squamous cell lung cancer glucose consumption that functions by inhibiting FLT3 and limiting Hexokinase 1 expression.

## Materials and methods

### Cell lines

SK-MES-1 (HTB-58, RRID:CVCL_0630), H520 (HTB-182, RRID:CVCL_1566), and H596 (HTB-178, RRID:CVCL_1571) cells were purchased fresh from ATCC and cultured in RPMI1640 supplemented with fetal bovine serum (10% v/v), glutamine (4 mM), and penicillin–streptomycin (100 U/mL). Cell lines were previously authenticated by ATCC.

### Plasmids

Nuclear-localized blue-fluorescent protein (BFP), Hexokinase 1, Hexokinase 2, JAK2, and FLT3 expression plasmids and an empty expression plasmid were from the University of California, Los Angeles Molecular Screening Shared Resource (UCLA MSSR). The FLIPglu-700 FRET plasmid was from Bernard Ribalet. BFP was transduced into cells using lentivirus. Cells were transfected with all other plasmids using FuGENE HD transfection reagent following the manufacturer’s protocol.

### High-throughput glucose consumption assay

The high-throughput glucose consumption assay was conducted as previously described^18^. Briefly, compounds were pinned into media (25 µL) in wells of a 384-well plate after which BFP-labelled cells (25 µL; 0.26 × 10^6^ cells/mL) were added. Cells were incubated (37 °C, 5% CO_2_, 24 h), wash three times with 1 × phosphate-buffered saline (PBS) containing 0.25% (w/v) bovine serum albumin (BSA), and 2-DG (1.25 mM final concentration) was added. Fluorescence in each well was imaged, cells were lysed, and detection reagent was added that converts 2-DG-6-phosphate levels to a light output. After 1 h, luminescence in each well was imaged. Compounds were incubated for 24 h unless otherwise noted.

### Z-factor determination

SK-MES-1, H520, and H596 cells were plated at a density of 0.26 × 10^6^ cells/mL in wells of a 384-well plate, incubated overnight (37 °C, 5% CO_2_), washed three times with 1 × PBS containing 0.25% (w/v) BSA, and treated with dimethyl sulfoxide (DMSO) or Cytochalasin B (10 μM). Glucose consumption was analyzed using our high-throughput glucose consumption assay except that 2-DG was not added to one set of cells. The *Z*-factor was calculated using the following formula, where µ_s_ and σ_s_ represent the mean and standard deviation of the positive control, respectively, which in our case is DMSO-treated cells, and µ_c_ and σ_c_ represent the mean and standard deviation of the negative control, respectively, which in our case is Cytochalasin B-treated cells or cells without 2-DG added: $Z=1-\frac{3({\sigma }_{s}+{\sigma }_{c})}{\left|{\mu }_{s}-{\mu }_{c}\right|}$.

### Compounds

The Prestwick library of mainly FDA-approved compounds, the Selleck Chemicals kinase inhibitors library, the LOPAC library of pharmacologically active compounds, and the NIH clinical collection were used for the high-throughput screen and were obtained from the UCLA MSSR. Individual compounds were purchased from Selleck Chem. Compounds were used at 10 µM concentration unless otherwise noted.

### High-throughput screen

SK-MES-1, H520, and H596 cells were screened against the described compound libraries and glucose consumption was measured using our high-throughput glucose consumption assay.

### [^14^C]2-DG accumulation

SK-MES-1, H520, and H596 cells were treated with vehicle or pacritinib for 24 h. Cells were washed 3 × with 1 × PBS. [^14^C]2-DG (5 µCi, 1.25 mM in 1 × PBS) was added to the cells, and the cells were incubated (30 min, 37 °C, 5% CO_2_), washed 3 × in cold 1 × PBS, and lysed in RIPA buffer. Lysate was added to scintillation fluid, accumulated radioactivity was measured on a scintillation counter, and this value was normalized to cell numbers.

### [^18^F]FDG accumulation assay

SK-MES-1 and H520 xenografts were grown in female, 10-week-old NOD *scid* gamma mice until they reached ~ 500 mm^3^. We used only female mice in these experiments as we have never identified differences in xenograft [^18^F]FDG accumulation between male and female mice^18^, and female mice are less likely to fight. [^18^F]FDG PET/CT imaging was conducted as previously described^18^ before and 24 h after treatment with vehicle (0.5% carboxymethylcellulose) or pacritinib (150 mg/kg; PO). Mice were assigned to the vehicle or pacritinib treatment groups at random. Accumulated radioactivity in the tumor and other organs was determined using the Amide Medical Imaging Data Analysis software by an individual unaware of the treatment groups. No mice were excluded from the analysis.

### FRET

The FRET assay was performed as previously described^18^ except that SK-MES-1 and H520 cells were used, and the cells were treated with vehicle or pacritinib.

### Immunoblots

Immunoblots were conducted as previously described^18^. The following antibodies were used: FLT3 (Cell Signaling Technology Cat# 3462, RRID:AB_2107052, validated through overexpression (in this work) and knockdown studies^45^); JAK2 (Cell Signaling Technology Cat# 3230, RRID:AB_2128522, validated through overexpression (in this work) and knockdown studies^46^); GLUT1 (Millipore Cat# 07–1401, RRID:AB_1587074, validated through overexpression^18^ and knockdown studies^47^); GLUT3 (Abcam Cat# ab15311, RRID:AB_301846, validated through overexpression studies^18^); Hexokinase 1 (Cell Signaling Technology Cat# 2024, RRID:AB_2116996, validated through overexpression (in this work) and knockout studies^48^); Hexokinase 2 (Cell Signaling Technology Cat# 2867, RRID:AB_2232946, validated through overexpression (in this work) and knockout studies^48^); β-actin (Cell Signaling Technology Cat# 4970, RRID:AB_2223172, used as a loading control in various studies^49–51^).

### qRT-PCR

qRT-PCR was conducted as previously described^18^. Briefly, RNA was isolated from treated cells using a GeneJET RNA Purification Kit (Thermo Fisher) per the manufacturer’s protocol. RNA (1 μg) was reverse transcribed to cDNA using the SuperScript First Strand cDNA Synthesis System (Thermo Fisher) per the manufacturer’s protocol. cDNA (5 ng per reaction, assuming quantitative conversion of RNA to cDNA) was amplified using the PowerUp SYBR Green Master Mix (Thermo Fisher) and the following primer sets. mRNA levels were normalized to β-actin mRNA levels.

#### GLUT1 forward:

GATTGGCTCCTTCTCTGTGG.

#### GLUT1 reverse:

 TCAAAGGACTTGCCCAGTTT.

#### GLUT3 forward:

GTCTGAAGAGCTATGGCCGC.

#### GLUT3 reverse:

AACCGCTGGAGGATCTGCTT.

#### Hexokinase 1 forward:

GGACTGGACCGTCTGAATGT.

#### Hexokinase 1 reverse:

ACAGTTCCTTCACCGTCTGG.

#### Hexokinase 2 forward:

CAAAGTGACAGTGGGTGTGG.

#### Hexokinase 2 reverse:

GCCAGGTCCTTCACTGTCTC.

#### Beta Actin forward:

TCACCCACACTGTGCCCATCTACGA.

#### Beta Actin reverse:

CAGCGGAACCGCTCATTGCCAATGG.

### Data analysis

All values were normalized to DMSO-treated or control overexpression cells. Data is plotted as mean ± standard error of the mean (SEM). Analyses were conducted and graphs plotted in Graphpad Prism (Version 9.3.0).

### Statistics

Values were compared using unpaired *T* tests and one- and two-way ANOVA analyses with multiple comparison testing for all experiments except the PET assay. There, a paired *T* test was used to compare [^18^F]FDG accumulation in the same mouse pre- and post-treatment.

### Approvals

All animal experiments were approved of by the UCLA Institutional Animal Care and Use Committee and performed in accordance with the Public Health Service Policy on Humane Care and Use of Laboratory Animals and the ARRIVE guidelines.

## Supplementary Information

Supplementary Information.

## Data Availability

All data used to generate this manuscript is presented in the figures, supplementary figure, and supplementary table.
