# Supplementary material for: Pacritinib inhibits glucose consumption in squamous cell lung cancer cells by targeting FLT3
Source: Sci Rep. 2023 Jan 25;13:1442. doi: 10.1038/s41598-023-28576-2 (PMC9876922; doi:10.1038/s41598-023-28576-2)
Supplement: Supplementary file 1 — Supplementary Information. [file 41598_2023_28576_MOESM1_ESM.pdf]

Supplementary Material for

Pacritinib inhibits glucose consumption in squamous cell lung cancer cells by targeting FLT3

Chiara Ghezzi, Bao Ying Chen, Robert Damoiseaux, Peter M. Clark

Supplementary Figures 1 – 3
Supplementary Table 1

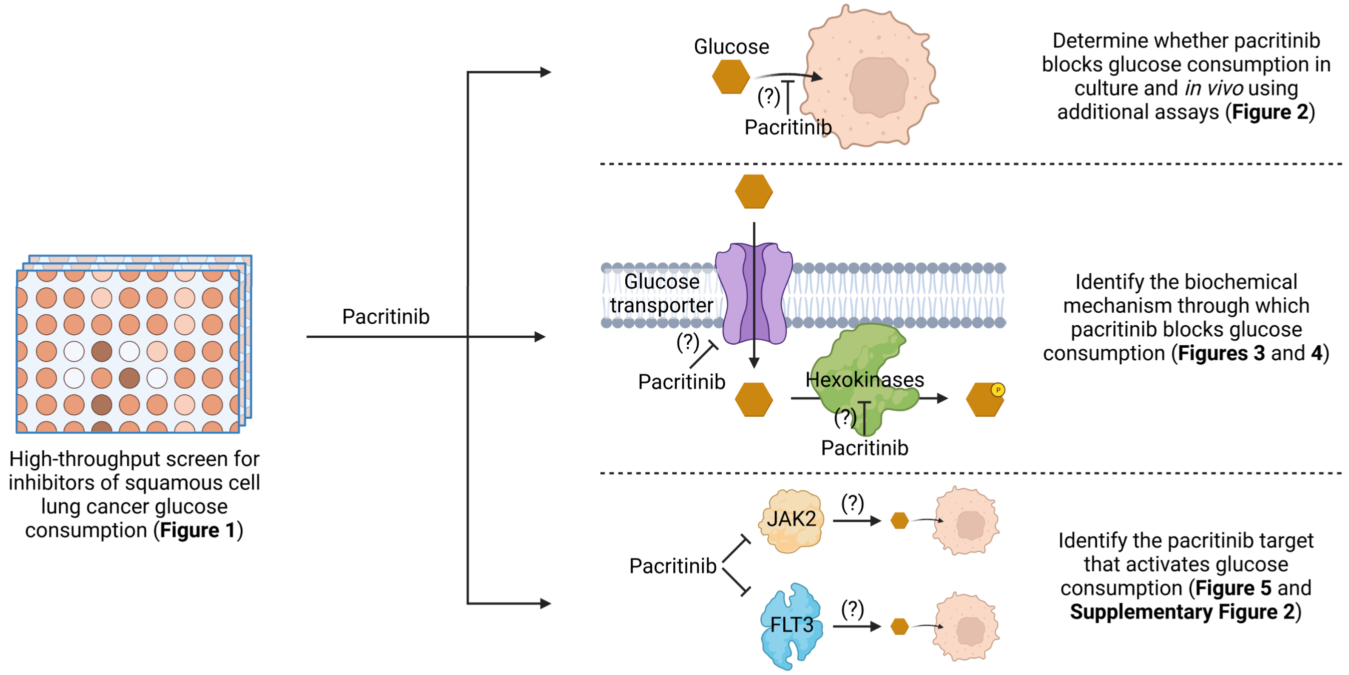

Supplementary Figure 1. Flow chart of the experimental design.

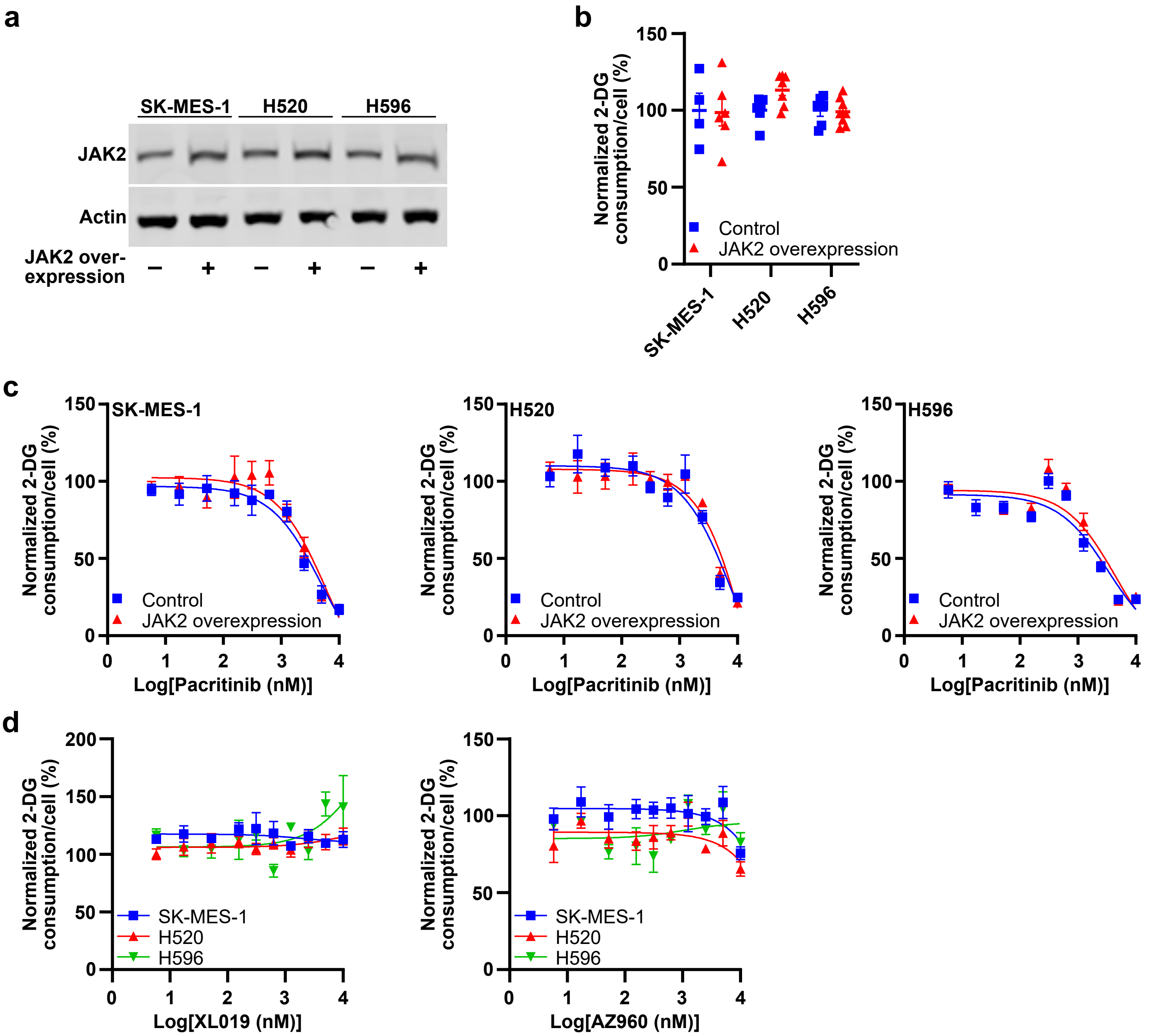

Supplementary Figure 2. Pacritinib does not block glucose consumption through targeting JAK2. a. Representative immunoblots of JAK2 in SK-MES-1, H520, and H596 cells transfected with control or JAK2 overexpression plasmids. Full-length, uncropped blots are presented in Supplementary Fig. 3. **b.** Glucose consumption in control and JAK2 overexpression SK-MES-1, H520, and H596 cells. **c.** Glucose consumption in control and JAK2 overexpression SK-MES-1, H520, and H596 cells treated with vehicle or pacritinib. **d.** Glucose consumption in SK-MES-1, H520, and H596 cells treated with vehicle or additional JAK2 inhibitors.

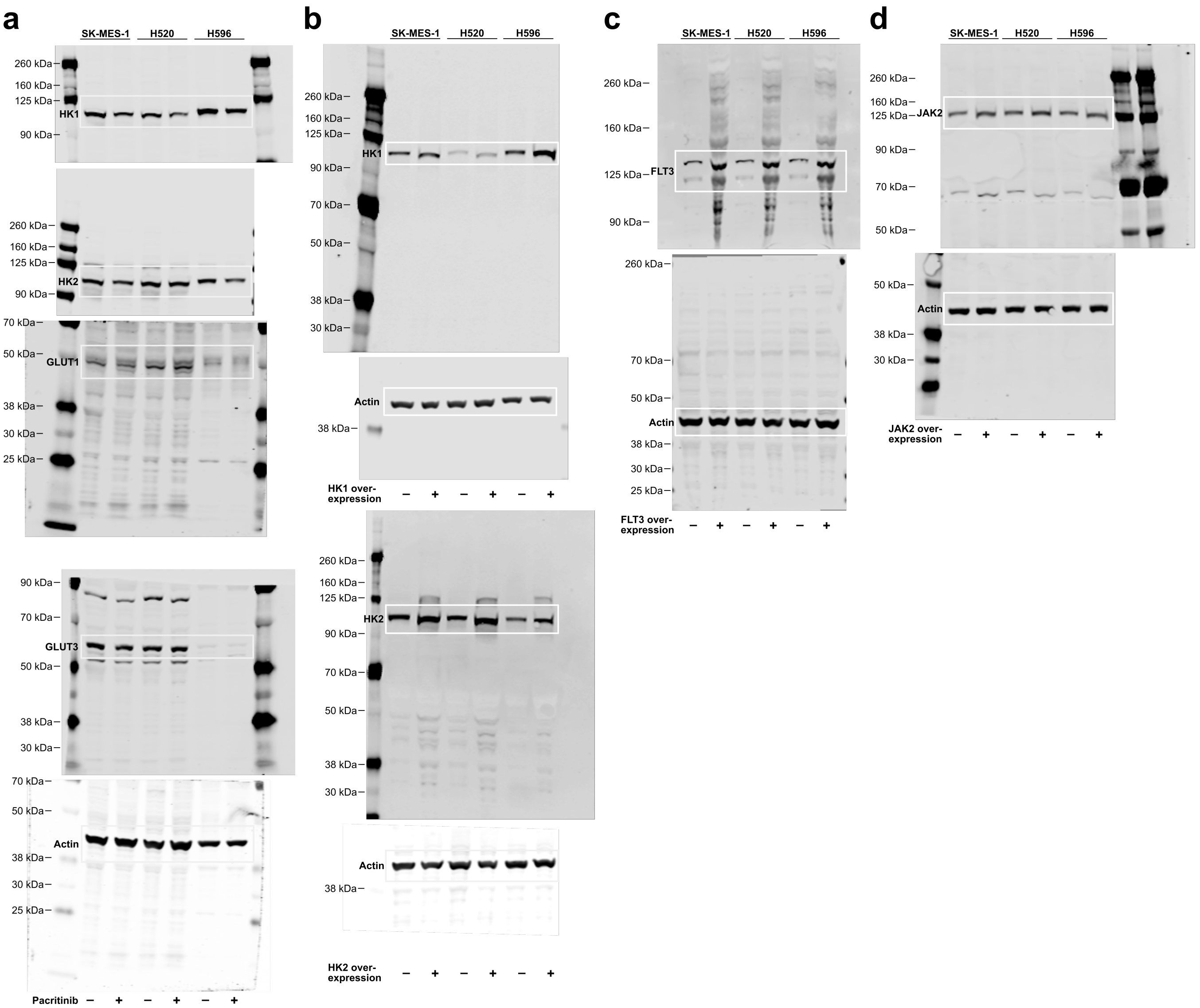

Supplementary Figure 3. Full-length, uncropped blots for (a) Fig. 3b, (b) Fig. 4a, (c) Fig. 5a, and (d) Supplementary Fig. 2a.

Supplementary Table 1: Small molecule inhibitors of glucose consumption in each of the tested squamous cell lung cancer cell lines

SK-MES-1
6-Methyl-2-(phenylethynyl)pyridine
α–Santonin
AC-93253 iodide
AT7519
AZD2014
AZD5438
AZD8055
BGT226
BKM120 (Buparlisib)
BMS-536924
Brefeldin A
Calcimycin
Camptothecine (S,+)
CHIR-124
CUDC-101
CUDC-907
Dorsomorphin 2HCl
Dovitinib (TKI-258)
Entrectinib (RXDX-101)
Idarubicin
KU-0063794
LY2835219
Mitoxantrone
MK-2206
Naringin
OSU-03012 (AR-12)
P276-00
Pacritinib (SB1518)
PD 180970
PD173955
Ponatinib (AP24534)
PP121
SGI-7079
Thapsigargin
Topotecan
Torin 2
Trifluoperazine
WYE-354

H520
Apomorphine
Auranofin
AZD2014
Brefeldin A
Brequinar
Camptothecine (S,+)
CUDC-907
Danshensu
Dihydroouabain
Ellipticine
Idarubicin
Mitoxantrone
MS-1502032
MS-1502111
Naringin
Niclosamide
Nitidine chloride
P276-00
Pacritinib (SB1518)
Ponatinib (AP24534)
Sanguinarine chloride
SC1
Securinine
SIB 1893
Topotecan

H596
α–Santonin
Akti-1/2
Apigenin
AZD2014
AZD3463
AZD5363
AZD8055
BKM120 (Buparlisib)
BMS-536924
Bosutinib
CH5132799
CHIR-124
Dacomitinib (PF299804)
Dipyridamole
Dorsomorphin
Dovitinib (TKI-258)
Entrectinib (RXDX-101)
GDC-0941
GSK2126458 (GSK458)
IMD 0354
INK 128 (MLN0128)
KU-0063794
LY3023414
MK-2206
Naringin
Nintedanib (BIBF 1120)
Nitidine chloride
Ofloxacin
Ouabain
Pacritinib (SB1518)
PD-166285
PD-180970
PD173074
PD173955
PI-103
Ponatinib (AP24534)
Pyrvinium pamoate
Quizartinib (AC220)
Rapamycin (Sirolimus)
Rifampicin
SB-366791
Thapsigargin
Torin 2
Triciribine
VS-5584 (SB2343)
WYE-125132 (WYE-132)
